# Studies of Auxetic Structures Assembled from Rotating Rectangles

**DOI:** 10.3390/ma17030731

**Published:** 2024-02-03

**Authors:** Julian Plewa, Małgorzata Płońska, Grzegorz Junak

**Affiliations:** 1 Faculty of Science and Technology, Institute of Materials Engineering, University of Silesia in Katowice, 75 Pułku Piechoty Str. 1a, 41-500 Chorzów, Poland; malgorzata.plonska@us.edu.pl; 2 Faculty of Materials Engineering, Silesian University of Technology, 44-100 Gliwice, Poland; grzegorz.junak@polsl.pl

**Keywords:** auxetics, Poisson’s ratio, rotating units cells, expansion

## Abstract

The subject of the work is analysis, which presents a renowned auxetic structure based on so-called rotating polygons, which has been subject to modification. This modification entails introducing pivot points on unit cell surfaces near rectangle corners. This innovative system reveals previously unexplored correlations between Poisson’s ratio, the ratio of rectangle side lengths, pivot point placement, and structural opening. Formulas have been derived using geometric relationships to compute the structure’s linear dimensions and Poisson’s ratio. The obtained findings suggest that Poisson’s ratio is intricately tied to the structure’s opening degree, varying as the structure undergoes stretching. Notably, there are critical parameter limits beyond which Poisson’s ratio turns positive, leading to the loss of auxetic properties. For elongated rectangles, extremely high negative Poisson’s ratio values are obtained, but only for small opening angles, while with further stretching, the structure loses its auxetic properties. This observed trend is consistent across a broad category of structures comprised of rotating rectangles.

## 1. Introduction

Metamaterials are man-made materials with unusual optical, electrical, or magnetic properties with no counterparts in nature. They can be regarded as a new class of ordered materials whose properties stem from qualitatively new functions in response to the applied stimuli. These properties are not observed in the constituent materials but result from the arrangement of the structural units into homogenised continua. One can, therefore, think of metamaterials as artificial materials produced by the appropriate spatial arrangement of so-called unit cells. Within the group of metamaterials with specific mechanical properties, one can further distinguish those with auxetic properties and those with non-auxetic properties.

Auxetic structures are mechanical, physical structures that, when subjected to external forces, exhibit simultaneous changes in their linear dimensions in the longitudinal and transverse directions. In tension, they expand longitudinally and transversely, whereas the reverse is true in compression. In physical terms, they constitute a continuum made up of repeating structural units that, when linked, transmit a force that moves the structure. The synthetic parameter for auxetic structures is Poisson’s ratio, understood as the ratio of the relative change in linear dimensions in the longitudinal and transverse directions. It is, therefore, related to the internal movement of structural units. An auxetic structure exhibits a negative Poisson’s ratio when moving from position 1 to position 2, the former of which is usually the initial one.

Among the best-known structural units of auxetic structures are the so-called rotating polygons (squares, rectangles, triangles) first proposed by Retsch [1,2] in 1965. However, it is only since 2000 that Grima has popularised them as auxetic structures [3,4,5].

It is noteworthy that among the notable auxetic structures are those whose elementary cells are of the re-entrant type and the chiral type. The former (re-entrant) are derived from Almgren [6], while chiral cells were first presented by Wojciechowski [7].

Numerous publications can be found on auxetic structures made up of rotating squares [3,8,9,10,11,12], rotating rectangles [4,13,14,15], and other rotating polygons [5,16]. Among the numerous theoretical studies of structures made of rectangular structural units, the work of Grim’s team should be singled out, in which their auxetic properties were demonstrated for a flexible combination of structural units [4,17].

A number of publications on these metamaterials introduce modifications to their structure. These include building composite systems [18,19,20,21,22] or hierarchical systems [10,23,24,25,26] or modifying the connections of the rotating polygons [21,27]. A distinct portion of research concerns the fabrication of rotating polygon structures by cutting out rhombuses from selected materials (perforated plate): polymer [12,28,29] and textiles [30]. In this case, auxeticity is achieved, although the square unit cells are not quite rigid. Generally known structures made up of rotating polygons are characterised by their internal geometrical structure, which consists of a flexible part (joints) and a rigid part (unit cells). The flexible part allows the basic structure to rotate or move, while the rigid part constrains the flexible part and provides the framework to maintain structural stability.

The flexible joints in the structure can only change in their dimensions and shape within the range of elastic elongation, which usually does not exceed a few per cent, although, for polymers, it can reach higher values. It is well known that as a result of the applied load, especially when applied cyclically, the joints of the unit cells become damaged, and the structure loses its properties.

One remedy for this inconvenience is to connect the unit cells with the pivot points (in the form of cylinders) on their corners. The idea of connecting the unit cells with cylinders is not new, e.g., Ref. [5], but the resulting relationships and well-functioning structures utilising it are not well-known. This solution—combining square or rectangular structural units with pivots—has become very appealing, and the resulting structures exhibit novel characteristics. A modified model of rotating polygons is thus obtained, demonstrating reliable and fail-safe functioning in tension and compression [31].

This is an improved two-dimensional version of the well-known ‘rotating squares’ model [1,3].

When such structures are stretched or compressed, the units not only rotate and change the angles between their adjacent sides, but they also shift, leading to planar expansion or contraction, which constitutes an auxetic behaviour. Such a structure deforms not only by the relative rotation of the squares but also by their translation.

The present work attempts to analyse auxetic structures based on real physical models made up of rectangles connected by pivots, with the geometric relationships as the main focus of the analysis. This approach allows us to immediately validate the mathematical relationships and determine their limits. Because of the modifications made to the rotating rectangles by introducing pivots connecting the unit cells, there have been particular constraints on the implementation of the structures. Namely, the angle of rotation of the units was constrained to a certain limit as a result of the compression of the structure, while the placement of the pivot points depended on the size of the rectangular cells.

The rectangles are connected by pivots and partially overlap with each other, exhibiting little friction (depending on the degree of roughness). A prerequisite for the proper functioning of the structure is the rigidity of the material of the rectangle units, which interact with each other through pivot points in tension or compression. In the closed position, however, parts of the edges of the rigid rectangles rest on each other and offer resistance.

This work attempts to bridge the gap between the multitude of simulation studies and the potential application of auxetic structures. Especially since, to achieve this goal, it is necessary to re-examine the results of the simulation studies and build real physical models, as well as verify their functioning. It is, after all, generally accepted that analyses concerning auxetic structures are carried out using simulation methods and computer graphics. They provide general information on these metamaterials, which, however, often requires revision based on the behaviour of real metamaterial structures. The need to verify and validate simulation results is usually neglected.

In the simulations, the mechanical metamaterial is not a tangible thing and is rather a result of the design approach. The study presented here, on the other hand, is concerned with physical structures for which the crucial characteristics and relationships between geometry and other properties have been identified. This aspect of the work is both innovative and practical.

## 2. Problem Statement

### 2.1. Modified Structure of ‘Rotating Rectangles’

**Figure 1:**
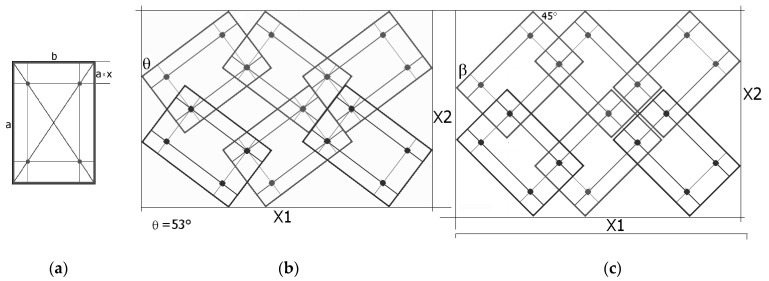
Rigid structural unit with marked pivots and the geometrical parameter x, and a metamaterial structure made of 4 rectangles connected by pivots with marked angle *θ* in the closed position and structure dimensions X1 and X2. In the figure: rectangle structural unit (**a**) where “a” and “b” are the lengths of the rectangle sides, the left structure (**b**) is in the *closed* position, and the right structure is in the *open* position (**c**).

The metamaterial structure is made up of units in the form of rectangles with side length ratios a/b. The rectangles are connected by pivots located on their diagonals at a distance of x × a from the edge of the shorter side b (Figure 1). By joining the units, a metamaterial structure is formed, the smallest segment of which contains four such units.

When an axial force is applied in a horizontal direction, the structure is simultaneously stretched, and the rectangles rotate about the pivots. The shear forces acting on the pivots depend on the applied tensile or compressive stress.

Structures of this type can be produced, granted that the geometrical parameter x satisfies the condition:

(1) $$x<\frac{1}{2(\frac{a}{b}+1)}$$

For a parameter value of x = 0.5/(a/b + 1), the structure becomes locked because the unit cells block each other.

In addition, it is also necessary to fulfil the second condition, which ensures the folding of the structure when it is compressed into the *closed* position. This position is characterised by the angle *θ*, whose value depends both on the placement of the pivot (parameter x) and on the size of the unit cell expressed by the a/b ratio.

**Figure 2:**
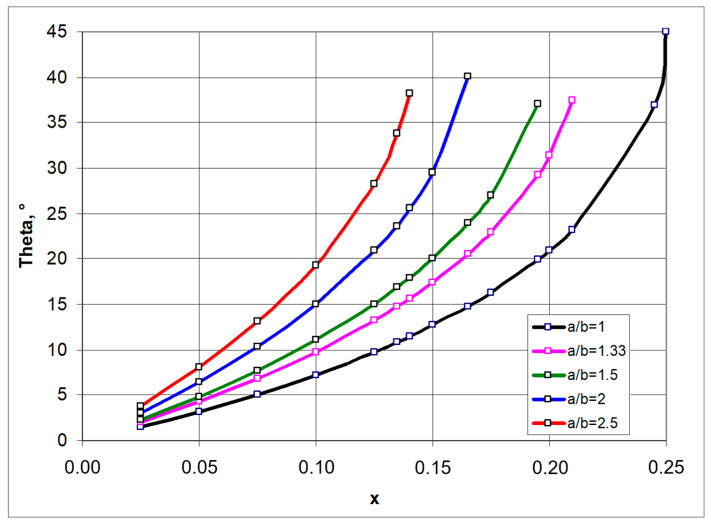
Relationship between theta angle (in the closed position) and the parameter x.

Figure 2 shows the required relationship between the angle *θ* and the x parameter for the given a/b values.

The relationship shown (Figure 2) means that, for a given value of the geometric parameter x, one can produce a structure made up of the so-called rotating rectangles with a side ratio a/b, which in the closed position reaches the specified angle *θ* (as in Figure 1b). The value of the angle *θ* is then used to determine the size of the structure in the closed position, providing the basis for calculating the Poisson’s ratio. On the other hand, the auxetic properties (negative Poisson’s ratio) of the structure are obtained for certain values of the parameters a/b and x and a corresponding opening of the structure by an angle β, with β > *θ*. The maximum elongation of the structure corresponds to the angle value β resulting from the dimensions of the structure:

(2) $$tan\beta_{max}=\frac{a}{b}$$

where a and b are the lengths of the rectangle sides.

When stretching the structure and moving from the closed to the open position, there is a change in the angle, which can reach the maximum value resulting from the condition (2).

**Figure 3:**
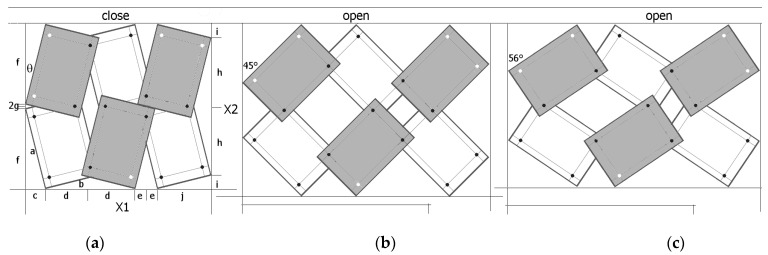
3 × 2 structure in the *closed* position (**a**) and two *open* positions for β = 45° (**b**) and for β = 56° (**c**), for a/b = 1.5, with marked lengths relevant for calculating the dimensions.

The deformation mechanisms and the value of the Poisson’s ratio can be readily identified by visual analysis of the structure. Figure 3 contains the marked and compared sizes X1 and X2.

In the case shown in Figure 3, the dimensions of the structure are calculated as the sum of the component lengths.

(3) X1=c+2d+2e+j and X2=2i+2h

The Poisson’s ratio is determined from the following relationship derived from axial displacement:

(4) $$\nu_{12}=-\frac{\frac{X1(open)-X1(close)}{X1(close)}}{\frac{X2(open)-X2(close)}{X2(close)}}$$

In contrast, the value shown below ν_21_ is the inverse of ν_12_.

The Poisson’s ratio accounts for the changes in the dimensions of the structure, i.e., it is related to the displacement caused by uniaxial tension or compression. In this sense, it is the so-called engineering Poisson’s ratio [5], defined as the negative ratio of the relative linear strains in which the initial and final states of the system are relevant.

In the case of the modified ‘rotating rectangles’ structure under consideration, the Poisson’s ratio depends on the parameters a/b and x or a/b and *θ*, while for a given parameter pair, it does not depend on the number of unit cells (rectangles) in the structure.

Referring to Figure 3, the considered system can be represented by a set of periodically arranged unit cells in which rotating rectangles with overlapping corners are placed in two adjacent planes. Thanks to this alignment, the projections of the structure on the plane allow us to find the formulae for calculating the dimensions of the structure. It can be added that the geometry of the structure is quite complex, and the value of the Poisson’s ratio can be obtained by deriving analytical equations involving the geometrical parameters of the structure.

To determine the structure dimensions of X1 and X2, in both the closed and open positions, the following relationships are used for the component lengths: where a and b are the lengths of the rectangle sides, x is the geometric parameter defined in Figure 1, and the angle *θ* is the angle between the axis X2 and the longer edge of the rectangle in the closed position. The section lengths listed in Equation (5) were defined according to Figure 3a. This angle is characteristic of the closed position and is a function of the size of the rectangle and the parameter x. Upon stretching of the structure, as the rectangles shift, this angle increases until it reaches the value of the rectangles’ diagonal angle (maximum opening position)—Formula (2).

Such a structure is deformed only by the relative rotation of the rectangles and the component lengths (Equation (5)), and hence, the structure dimensions X1 and X2 are functions of a single variable—the changing angle.

(5) $$c=a\times \sin\theta \;d=(b-bx-ax\tan\theta )\times \cos\theta \;e=(a-ax-\frac{bx}{\tan\theta })\times \sin\theta \;f=a\times \cos\theta \;g=(b-bx-\frac{ax}{\tan\theta })\times \sin\theta \;h=(a-ax-bx\tan\theta )\text{×}\cos\theta \;i=b\times \sin\theta \;j=b\times \cos\theta$$

### 2.2. Analytical Models Based on Geometric Transformations

The analysis of structures of arranged polygons can be carried out using simple rules derived from trigonometry. The regular arrangement of the unit cells makes it easy to find the relationship between the lengths of the individual sections and the angles between them. Such an analysis yields reliable results, unhindered by any simplifying assumptions.

#### 2.2.1. Properties of a 4 × 4 Structure (for a Fixed Value of x)

**Figure 4:**
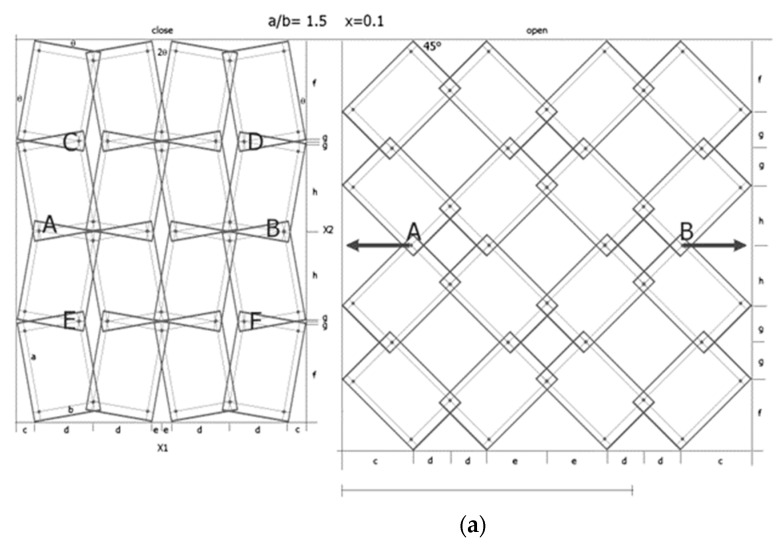

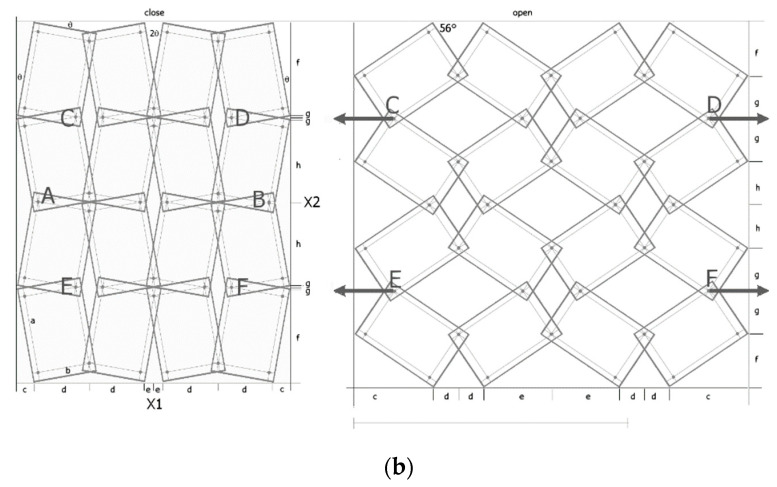
4 × 4 structure in the *closed* position and two *open* positions, for a/b = 1.5 x = 0.1, *θ* = 11.04° with marked lengths for calculating the dimensions of the structure.

The structures chosen for the analysis are 4 × 4 structures made up of rectangular unit cells with a/b = 1.5 for the value of the geometric parameter x = 0.1, at which the auxetic properties (ν_12_ < 0) are obtained. Both the opening of the structure up to an angle of 45° as well as its opening at the maximum elongation (β = 56°) lead to negative Poisson’s ratio values (Figure 4).

A structure under tension in the horizontal direction causes a change in the inclination of each parallelogram relative to the plane of the structure’s surface. This results in the opening of the entire structure, leading to increased dimensions in both the horizontal and vertical directions and the NPR (Negative Poisson’s Ratio) effect.

When stretching a structure with the parameter x = 0.1, rectangles of size a/b = 1.5 rotate from the angle *θ* (closed position) to a maximum angle β = 56°. In the open position for the angle β = 45°, maximum expansion is only achieved for square units (a/b = 1). In the example analysed (Figure 4), the transition from the closed position to an open position results in a Poisson’s ratio of −6.29 and −171.1 for β = 45° and β = 56°, respectively. It may be added that the observed expansion cannot be initiated internally and, in order to expand the system, external tensile force must be applied to the pivots at points A and B in order to achieve the angle β = 45°, while in order to achieve the angle β = 56°, tensile force must be applied at points C, D and E, F, as shown in Figure 4. This means that when the structure is fully stretched (β = 56°), the diagonals of the rectangles align with the direction of stretching. The structure expands as it is stretched (uniaxially), utilises the uniformity of the rigid elements, and, in practice, only demonstrates reliability for pivot connections.

It is obvious that if the deformation of the rectangles or shearing of the pivots occurs, the structure will lose its auxetic properties. The pivot material must have good mechanical properties, particularly a good shear resistance. The presented solution allows full control in terms of deformation and makes it possible to reproduce the auxetic behaviour at various scales.

Using the relationships given above, the characteristic values of Poisson’s ratio and relative elongation in tension were calculated for the analysed structure. For the 4 × 4 structure shown in the figure, the auxetic properties are obtained.

**Figure 5:**
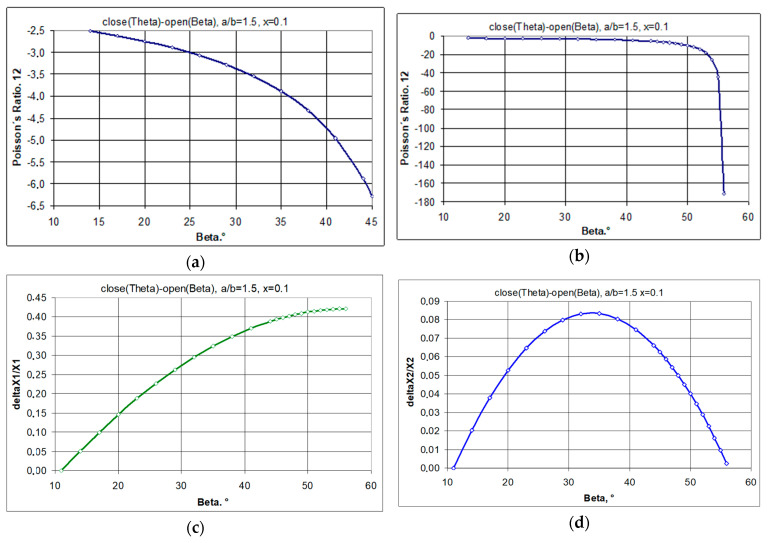
Poisson’s ratio and relative elongation as a function of opening angle β for the 4 × 4 structure, with a/b = 1.5 and x = 0.1, (**a**)—ν_12_ vs. β for the forces applied at points A and B, (**b**)—a—ν_12_ vs. β for the forces applied at points C, D, E and F, (**c**)—ΔX1/X1 vs. β, (**d**)—ΔX2/X2 vs. β.

The illustrations (Figure 5) show how the Poisson’s ratio ν_12_ changes with the stretching of the 4 × 4 structure when tensile forces are applied at points A and B and the resulting opening angle reaches 45°, and when the forces are applied at points C, D and E, F causing a final opening with beta = 56° (see Figure 4). In the latter case, very high NPR values are achieved. It should be noted that for higher values of ν_12_, ν_21_ tends towards very small values. This is a consequence of minor dimensional changes.

Characteristics of the 4 × 4 structure, with a/b = 1.5 and x = 0.1, as shown in Figure 5, indicate the full auxetic properties achieved. The negative value of the Poisson’s ratio increases with the degree of opening of the structure. When stretched, the structure expands horizontally in a monotonic manner, while vertically, it initially expands, reaching a maximum, yet after that, it contracts. If the elongation reaches very small (yet still positive) values, the negative Poisson’s ratio increases rapidly. This occurs for large values of the degree of opening, β > 50°.

The relationships presented here most importantly indicate that Poisson’s ratio is always a function of the degree of opening of the structure. Its value changes when the structure is stretched.

#### 2.2.2. Properties of the 4 × 4 Structure (for Different Values of x)

If, for the structure shown in Figure 4, the position of the pivot point is changed, i.e., the geometrical parameter x is altered, one can produce various metamaterial structures, both auxetic and non-auxetic.

By changing the position of the pivot points, with the geometrical parameter x varying within the allowed limits, the Poisson’s ratio varies within wider limits.

**Figure 6:**
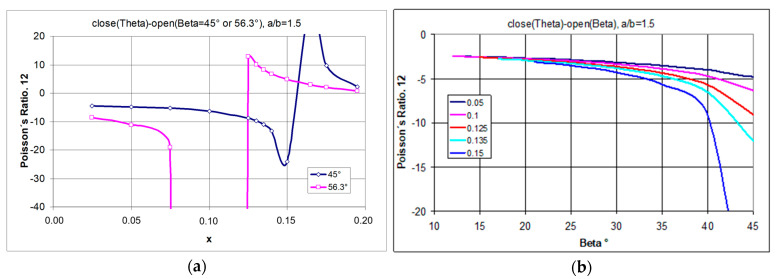
The change in Poisson’s ratio for two open positions, structure with a/b = 1.5 for different values of the geometrical parameter x, (**a**)—ν_12_ vs. x, (**b**)—ν_12_ vs. β.

By first considering the maximum stretching of the structure (from closed to open position), the relationship between Poisson’s ratio and the geometric parameter x was derived (Figure 6a). For a selected rectangle with a side ratio a/b = 1.5, one obtains greater values of the Poisson’s ratio the greater the parameter x, i.e., the further the pivot point is from the edge of the rectangle. Whereas for specific values of the parameter x (x → 0.1 or x → 0.16), extremely high NPR values are obtained. However, these values relate to the high degree of opening of the structure—the high values of the angle β (Figure 6b).

The included calculation results show that by changing the position of the geometrical parameter x, one can switch between an auxetic and a non-auxetic behaviour (Figure 6a). By shifting the position of the pivot point of the rectangles, positive values of the Poisson’s ratio can be obtained (for large values of the angle β).

A further possibility of obtaining the desired NPR values for the structures under consideration is to change the dimensions of the rectangular unit cells and, in particular, the a/b ratio values. By manipulating the size of the rectangular cells, further possibilities are opened in the whole spectrum of structures with a high negative Poisson’s ratio.

**Figure 7:**
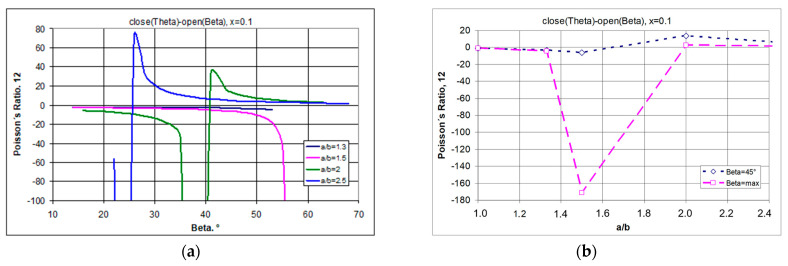
Changes of the Poisson’s ratio as a function of the opening angle for structures made of rectangles with different a/b (**a**) and Poisson’s ratio values as a function of the a/b parameter for the maximum opening (**b**). The angle of the maximum opening follows the relationship β = arctan(a/b).

The demonstrated relationships indicate that there is a limit to the strain (degree of opening) for which structures exhibit an auxetic behaviour (Figure 7a). This statement indicates that in producing modified auxetic structures using rectangles as unit cells (subject to the above-mentioned conditions), the auxetic properties can always be obtained if the angle β of the opening of the structure is sufficiently small.

It should be noted that structures (at x = 0.1) made of rectangles with a/b = 1.3 and 1.5 show auxetic properties for the whole range of the β angle, while those with a higher value of a/b (a/b > 1.5) first show negative and then positive values of the Poisson’s ratio (Figure 7b). Moreover, a particular property of such structures is that for a/b > 1.3, they can achieve extremely high values of the Poisson’s ratio.

**Figure 8:**
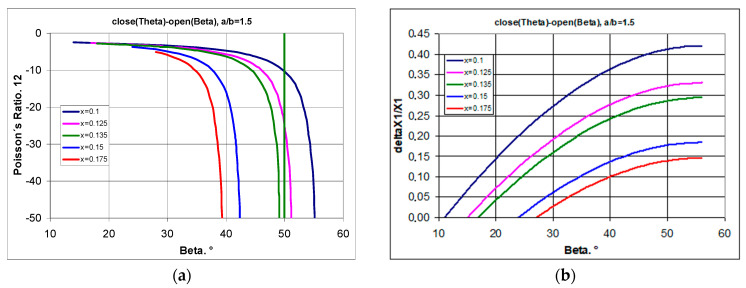
Change in Poisson’s ratio as a function of the opening angle of the structure for the range within which auxeticity is exhibited (**a**) and the magnitude of the expansion as a function of the opening angle (**b**) for structures a/b = 1.5.

By changing the position of the pivot points, the increase in the parameter x can also result in a significant change in the Poisson’s ratio, accompanied by a decrease in relative elongation. This property is shown in Figure 8.

The presented graphs (Figure 8) indicate that the analysed structure is able to exhibit negative Poisson’s ratios for large strains (with large changes in the opening angle β). One can also observe an increase in relative elongation along with the increase in the opening angle β, although this is limited by the increase in the parameter x. The further the pivot point is from the edge, the smaller the relative elongation (Figure 8b).

**Figure 9:**
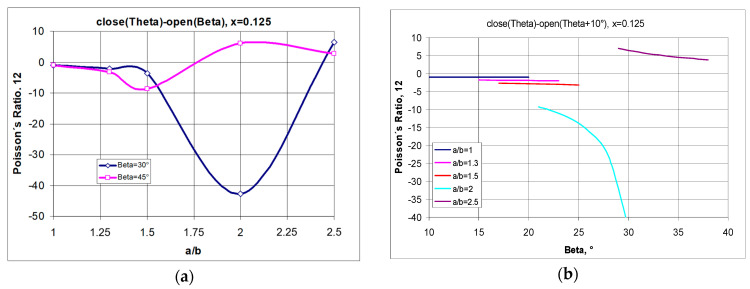
Relationship between Poisson’s ratio and the dimensions (a/b) of the rectangular cells for x = 0.125 when going from an angle of θ to the opening angle equal to 30° as well as to the angle of 45° (full opening)—(**a**), along with the Poisson’s ratio values for stretching individual structures (varying a/b) from an angle of θ + 10° (partial opening)—(**b**).

Regarding Figure 8**,** the system under consideration can be represented by a structure with x = 0.125. Considering the two extremes—in this case, the maximum elongation of the structure and, for comparison, small elongation—one can observe the particular tendencies presented in Figure 9.

From the equation in Figure 9a, it can be seen that a negative Poisson’s ratio can be obtained for structures made of rectangular units, provided that the opening angle is appropriately selected. For example, for a structure with the parameters a/b = 2 and x = 0.125, negative values can be obtained for the angle β = 30°, i.e., without stretching the structure to the limit value of 45°.

The results shown in Figure 9a for structures with x = 0.125 should also be addressed here. In this case, the negative Poisson’s ratio generally increases with the lengthening of the rectangles until they reach a maximum value, after which they move towards positive values. The effect is qualitatively similar for large and small degrees of opening, yet it is more pronounced for small values of the beta angle. It can be seen that when the structures are slightly stretched, for rectangles with the parameter value a/b = 2, there is a large negative change in the Poisson’s ratio (Figure 9b), while for shorter rectangles, the negative Poisson’s ratio values are not very large and almost constant when the structure is stretched from the angle *θ* + 10° to the angle β. On the other hand, for a highly elongated structure a/b = 2.5, even with a small opening, the Poisson’s ratio is positive—as shown in Figure 9b.

**Figure 10:**
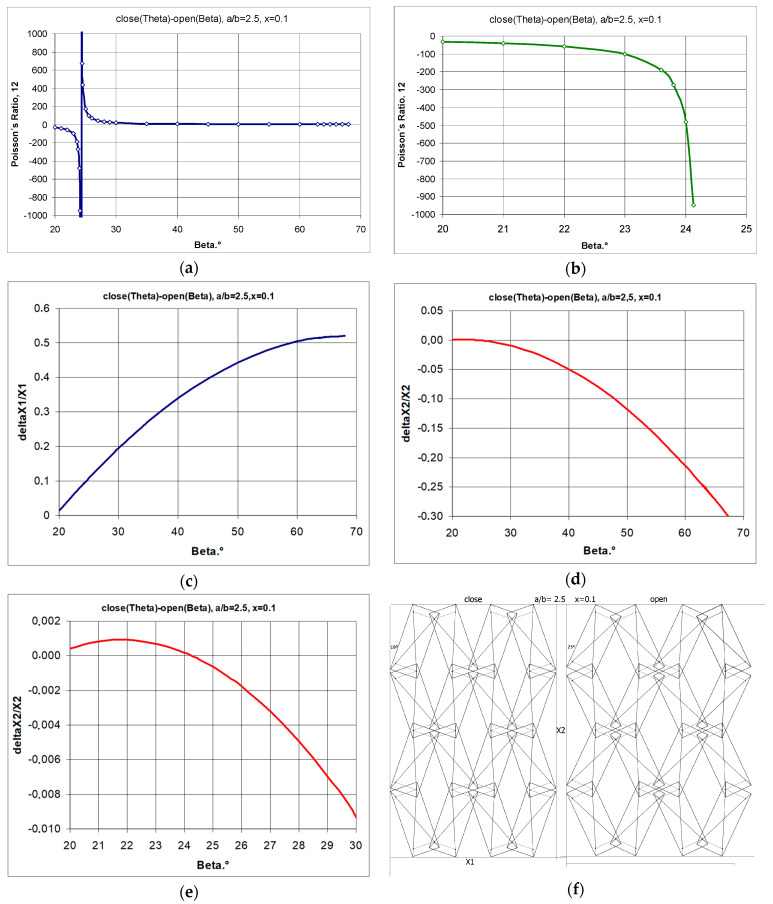
Changes in Poisson’s ratio as a function of the opening angle for small (**a**) and for large (**b**) values of parameter x—structure with a/b = 1.5 (where (**a**) ν12 vs. β; (**b**) ν21 vs. β; (**c**) ΔX1/X1 vs. β; (**d**,**e**) ΔX2/X2 vs. β; (**f**) structure sketch).

By manipulating the geometrical parameter x, or more precisely by reducing it, the auxetic properties can be obtained even for long rectangles. However, for structures made up of larger rectangles (a/b = 2.5), there is only a narrow range of auxeticity. This is determined by the low value of the parameter x and the small opening angle of the structure β. This is demonstrated in the example in Figure 10 with a structure that exhibits negative Poisson’s ratios at small strains and a positive Poisson’s ratio only when further stretched.

The structure shown in Figure 10f (a/b = 2.5) shows very limited auxetic behaviour and a tendency towards extreme values of the Poisson’s ratio.

The analysis obtained shows that structures with rectangles with a high a/b ratio obtain auxetic properties for small values of x and a small degree of opening β. After exceeding the limit value of the β angle (in this case, 24.25°—Figure 10a), the structures lose auxetic properties, with the increasing relative change in linear dimensions in the horizontal direction (Figure 10c), and the relative change in the vertical direction reaching a maximum and then strongly decreasing (Figure 10d,e). The graphical relationships in Figure 10 show that for an opening angle of 25°, i.e., above the structure’s limit value, a positive Poisson’s ratio is obtained. This example is intended to present the typical behaviour of metamaterial structures, which only exhibit auxetic properties in certain predefined conditions. As the presented structure allows for full control of the strain pattern, it can result in small elongations in the vertical direction at a high Poisson’s ratio, which may be of interest for applications in, e.g., electronics.

To demonstrate the possibilities of adjusting the parameters for a ‘rotating rectangles’ structure suitable for design purposes, a comparison was made as to how far a change in the position of the pivot points affects the value of the Poisson’s ratio.

**Figure 11:**
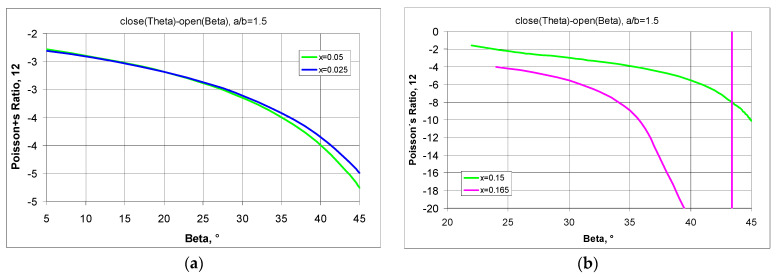
Changes in Poisson’s ratio as a function of the opening angle for small (**a**) and for large (**b**) values of parameter x—structure with a/b = 1.5.

The Poisson’s ratio for 4 × 4 structures with a/b = 1.5 in full elongation (*open* (Beta = 45°)) as a function of the parameter x, and the change in Poisson’s ratio values when stretched by the value of the Beta angle (Theta < Beta ≤ 45°) are shown in Figure 11.

Small values of x result in relatively small values of the Poisson’s ratio and with little change in it when the structure is maximally stretched (Figure 11a). On the other hand, for large values of the parameter x, as shown in Figure 11b, large changes in this ratio must be taken into account.

Depending on the needs, a desired auxetic structure can be designed from selected rectangles (with a/b ratio) by connecting the rectangles with pivots located according to the value of the geometrical parameter x.

To summarise the presented analysis of structures made of so-called ‘rotating rectangles’, one can be sure that it allows one to easily proceed to programming the fabrication of physical models and experimentally control the geometrical parameters in those models.

## 3. Models of Auxetic Structures

Flat auxetic structures made of rectangular unit cells show changes in linear dimensions when subjected to force. These changes are easy to follow on physical models, especially as their magnitude can be seen precisely on the basis of the analysis of structures outlined above.

In order to demonstrate that 2D mechanical metamaterials assembled from rectangles are able to exhibit a range of typical auxetic behaviours, numerous physical models were made. The assembly technique was used, connecting metal unit cells perforated near their corners. The precision of the manufacturing of the unit cells, i.e., the dimensions and the exact positioning of the perforations for the pivots, determines the correspondence between the theoretically calculated and measured dimensional changes of the assembled structures.

### 3.1. Examples of Conditionally Auxetic Structures

One can be convinced that actual physical structures can provide a better understanding of the potential of rotating rigid units to yield an auxetic behaviour.

#### 3.1.1. A 4 × 4 Structure Model, for a/b = 1.5 and x = 0.1

**Figure 12:**
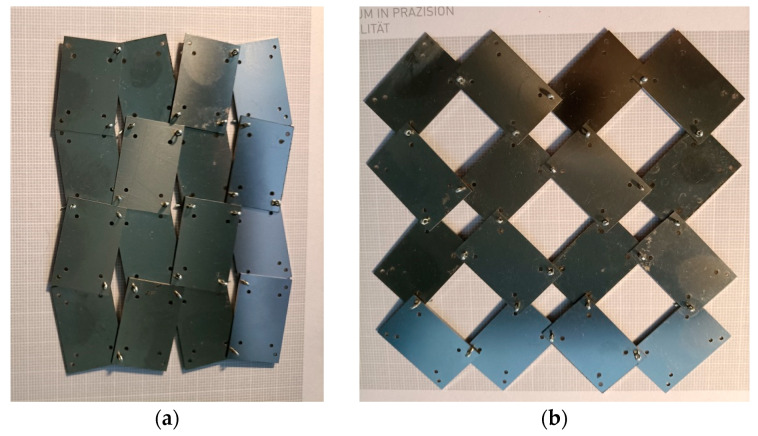
4 × 4 structure in *closed* (**a**) and *open* (**b**) positions.

Metal rectangular unit cells connected by pins are shown in Figure 12. For the given parameters a/b and x, the value of the theta angle is *θ* = 11.04°.

**Figure 13:**
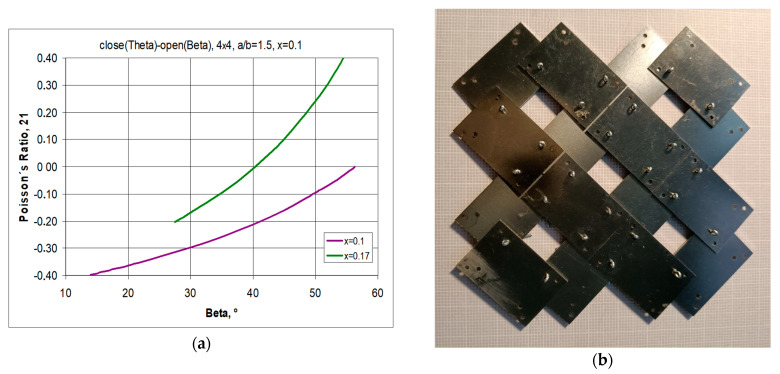
Relationship of the Poisson’s ratio as a function of the opening angle β for a structure with the parameters a/b = 1.5 and x = 0.1 and a/b = 1.5 and x = 0.17 (**a**), as well as a structure with the parameters a/b = 1.5 and x = 0.2 (**b**).

The transition between *closed* to *open* positions occurs when the structure is stretched horizontally. For symmetrical structures (same number of unit cells vertically and horizontally) stretched to β = 45°, a square shape is obtained, i.e., X1 = X2. For the given parameters a/b = 1.5 and x = 0.1, the structure exhibits full auxetic properties, i.e., NPR—Figure 13a. This applies to the stretching of the structure both up to an angle of β = 45° and up to a maximum stretch with an angle of β = 56.3°.

By manipulating the position of the pivots, structures can be obtained that exhibit both negative and positive Poisson’s ratio values. As shown in Figure 3a, increasing the value of the x parameter from 0.1 to 0.17 results in a structure exhibiting NPR below an opening angle of β < 40°. In contrast, the image of the structure with parameter x = 0.2 in Figure 13b shows unit cells mutually blocking each other. Such a locked structure is the result of the failure to fulfil condition (1).

#### 3.1.2. A 4 × 4 Structure Model, for a/b = 42/26 = 1.61, x = 5.5/42 = 0.1309, θ = 19°

**Figure 14:**
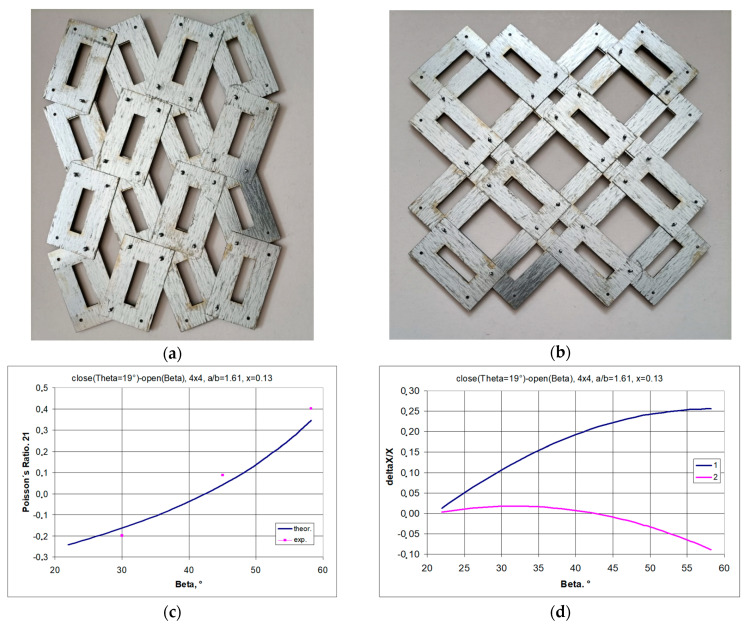
A 4 × 4 structure with parameters a/b = 42/26 = 1.61, x = 5.5/42 = 0.1309 in the *closed* position (**a**) and in the *open* position (**b**), along with the relationship of the Poisson’s ratio (**c**) with the relative change in linear dimensions (**d**) as a function of the opening angle β.

A structure constructed from rectangular frames with sides with a ratio of 1.61 and parameter x = 1.1309 is shown in Figure 14. It is immediately apparent that in the *open* position, the vertical dimensions of the structure are smaller than in the *closed* position. This means that for an angle of β = 45°, the structure has a positive Poisson’s ratio.

Also, for maximum elongation (β = 56.3°), the design does not achieve NPR. For angle values β < 40°, auxetic properties are exhibited (Figure 14c), which is due to the positive values of the relative dimensional changes (Figure 14d). As the structure stretches, it expands monotonically in the horizontal direction. Whereas the corresponding elongation of the structure in the vertical direction reaches a maximum: the dimensions in the vertical direction first increase and then decrease.

#### 3.1.3. A 10 × 4 structure Model, a/b = 25/12.5 = 2, x = 2.5/25 = 0.1, *θ* = 15°—Figure 15

**Figure 15:**
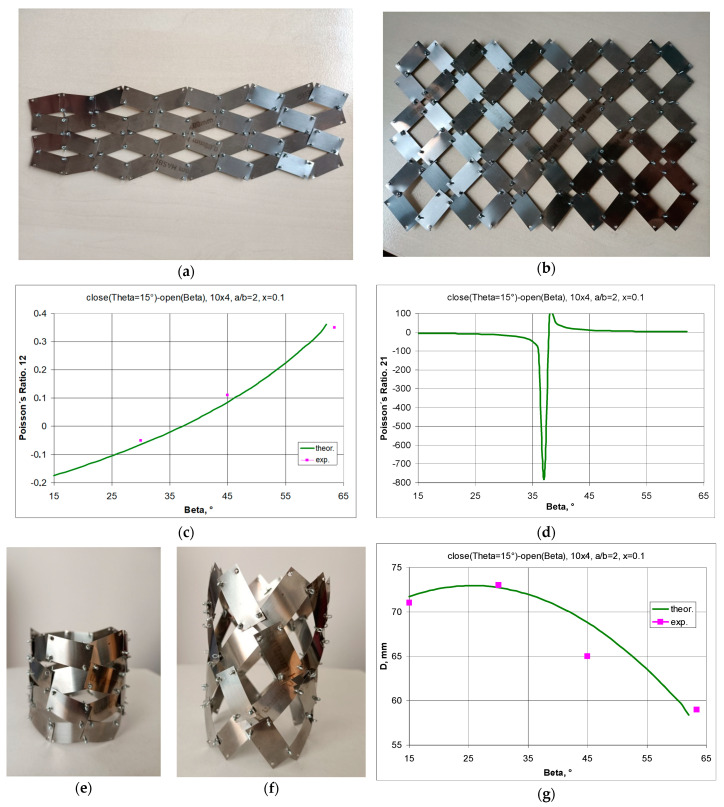
A 10 × 8 structure with the parameters a/b = 25/12.5 = 2, x = 2.5/25 = 0.1 in the *closed* position (**a**) and in the *open* position (**b**), and the Poisson’s ratio relationships 12 (**c**) and 21 (**d**) and the corresponding flat tubular structures in the *closed* position (**e**) and in the *open* position (**f**), as well as the change in diameter D of the tubular structure as a function of the opening angle β (**g**).

By using a rigid and flexible material (0.09 mm thick steel), a 2D structure was assembled from which a tubular structure could be produced. With the given parameters, a range of beta angles is obtained in which the structure exhibits NPR. This range is particularly evident for the Poisson’s ratio, both calculated as the ratio of the relative change in dimensions ΔX1/X1 to ΔX2/X2, i.e., ν12 (Figure 15c) and vice versa, i.e., ΔX2/X2 to ΔX1/X1, i.e., ν21 (Figure 15d). In the latter case, we are dealing with an extremely high Poisson’s ratio. This always corresponds to a very small change in linear dimensions in one of the directions.

The flat structure produces a tubular structure when rolled up (Figure 15e,f), whose auxetic behaviour only occurs for a diameter value D greater than that obtained for the closed position (i.e., β = 15°). Figure 15g shows the changes in the diameter of the tubular structure as a function of the opening angle β, both calculated theoretically and measured. The differences are due to minor gaps in the pivots and some inaccuracies in their positioning, which are common in the case of manual assembly.

## 4. Summary

The system analysed in this work is shown in Figure 1 and it can be described as an improved two-dimensional version of the well-known ‘rotating squares’ model. This improvement is achieved by introducing additional elements that create the possibility of rotating the rectangles. These additional elements are referred to in mechanical nomenclature as ‘pin joints’ and are used for linking and fixing the units. These are cylindrical units perpendicular to the structure of the rotating rectangles, which come closer together when the structure is compressed into the closed position. It can be shown that pin joints do not resist rotational movement and that the only resistance can arise from the friction of the overlapping parts of the rectangles. On the other hand, the shear force acting on the pin joints is transmitted through the rectangles. Its size corresponds to the friction resistance, which is generally low.

Thus, by solving the problem of stable combinations of rigid squares [28] and rectangles that retain their rotational motion by introducing the pivots at their corners, auxetic structures exhibiting large values of longitudinal and transverse linear expansion have been obtained. These expansion values are much greater than those occurring in unmodified solutions [9]. The applied solution, therefore, offers advantages over the earlier one known as ‘rotating squares’. It has been shown that to obtain a negative Poisson’s ratio, one has to adjust the geometrical parameters both concerning the elementary cells themselves (in this case, the size of the rectangle) and the placement of their pivots, which makes it easy to make structures of thousands of unit cells all having the corresponding properties. It is, therefore, possible to not only successfully predict the properties and theoretically design the structure but also successfully assemble it. The plots of Poisson’s ratio and elongation for different values of a/b and x shown in Figure 3–11 clearly demonstrate that the system modified by adding the pivots at the corners of the rectangular planes exhibits different properties. When the structure is compressed, the locking of the structural units in the so-called closed position, characterised by the angle *θ*, occurs. By adjusting the parameters a/b and x, the system can achieve ‘complete auxeticity’ in the sense of Wojciechowski, meaning that it can exhibit a negative Poisson’s ratio for the transition from *closed* to *open* positions. In this case, it is, for example, a/b < 1.5 for x = 0.1.

It was also shown that for the structures under consideration, the Poisson’s ratio values tend towards +∞ or −∞ when the structure is stretched beyond the limit values of the opening angle for specified parameters a/b and x. A special feature of the modified structure is the rigid structural units and the shear-resistant pin connections forming the pivots. As a result of the application of tensile and compressive forces, there is no hindrance to the rotation of the structure’s elements, as there is no elastic stress on the material.

In addition, the modified structures exhibit large dimensional changes because they do not exhibit the constraints that occur in unmodified structures containing joints. In these unmodified structures, the mechanical properties of the unit cell joints determine the size change, yet in most materials, the elastic range is relatively small (ε < 0.1).

The reliability and failure-free rotation of the rectangles during the stretching of the structure may also provide the basis for many practical applications that could involve electronic components in intelligent systems or structural designs. By adjusting the geometrical parameters a/b and x, one can precisely determine the range of Poisson’s ratio values and the desired changes in the size of the structure. It should be emphasised that the present work, using a modified system of ‘rotating squares’ [28] introduces a viable proposal for useful auxetic structures while being free of the constraints arising from the breaking of the joints as well as those related to their extensibility. Thus, it might be conducive to finding real-world application possibilities in the development of meta-devices. For example, as shown in Figure 4, impressive elongation effects can be achieved when stretching such a structure. In compression, but beyond the critical values, buckling of the structure can occur, which can be limited by the introduction of a sandwich structure. By linking several layers of unit cells, the structure becomes more stable and, at the same time, requires less material than an equivalent bulk structure. We, therefore, provide direct evidence of the reliability of the proposed system and its ability to achieve large negative Poisson’s ratios by directly measuring the deformations that occurred when the prototype shown in Figure 12 or Figure 15 was stretched.

The measurements taken confirmed the calculated changes in linear dimensions. In this respect, the work provides directions for the mass production of auxetic structures for various applications requiring a large negative Poisson’s ratio and a small elongation in the direction perpendicular to the force causing deformation of the structure.

## 5. Conclusions

Metamaterial structures assembled from rectangular structural units connected by pivots can exhibit both positive and negative Poisson’s ratio values (depending on the structural parameters and on the angle between the rectangles). The modified structures formed from square unit cells, on the other hand, can only exhibit a negative Poisson’s ratio of −1.

The modified concept of rotating rectangles has been tested experimentally, where the theoretically calculated constraints for its implementation have been confirmed. The structures presented here are, above all, feasible and differ markedly from the idealised models commonly found in research. Rectangles are treated as perfectly rigid units, and thanks to the introduced pivots, they rotate relative to each other without changing shape. The paper has presented the design and development of a modified metamaterial structure that can be applied in robotics. Its manufacturing involves linking rectangular unit cells with pivots. The assembly allows the manufacturing of structures with a large number of unit cells that can provide a defined degree of expansion. They can also serve as metamaterials for buckling and fracture mechanics studies. This is especially true since only physical models can allow us to understand the macroscopic properties of mechanical structures. Only the bulk behaviour of the metamaterial can provide evidence of its auxeticity and stability.

## Data Availability

Data are contained within the article.
